# The Renin–Angiotensin System in COVID-19: Can Long COVID Be Predicted?

**DOI:** 10.3390/life13071462

**Published:** 2023-06-28

**Authors:** Simone König, Richard Vollenberg, Phil-Robin Tepasse

**Affiliations:** 1 IZKF Core Unit Proteomics, University of Münster, 48149 Münster, Germany; 2 Department of Medicine B for Gastroenterology, Hepatology, Endocrinology and Clinical Infectiology, University Hospital Muenster, 48149 Münster, Germany; richard.vollenberg@ukmuenster.de (R.V.); phil-robin.tepasse@ukmuenster.de (P.-R.T.)

**Keywords:** mass spectrometry, RAS, ACE, ACE2, CPN, bradykinin, hypertension, cardiovascular disease, antihypertensive, ACE inhibitors, angiotensin receptor blockers, bradykinin, neuropeptide reporter assay

## Abstract

(1) Background: Co-morbidities such as hypertension and cardiovascular disease are major risk factors for severe COVID-19. The renin–angiotensin system (RAS) is critically involved in their pathophysiology and is counter-balanced by both angiotensin-converting enzyme 2 (ACE2), the functional receptor of SARS-CoV-2, and the kallikrein–kinin system (KKS). Considerable research interest with respect to COVID-19 treatment is currently being directed towards the components of these systems. In earlier studies, we noticed significantly reduced carboxypeptidase N (CPN, KKS member) activity and excessive angiotensin-converting enzyme (ACE, RAS member) activity in the sera of both hospitalized COVID-19 patients and a subgroup of convalescent patients. The data had been obtained using labeled bradykinin (BK) as a reporter peptide, which is a target of both CPN and ACE. The data were supplemented with mass-spectrometry-based serum proteomic analysis. Here, we hypothesize that the degree of BK serum degradation could be indicative of Long COVID. (2) Review and Discussion: The recent literature is briefly reviewed. The fact that the levels of the BK serum degradation products did not reach normal concentrations in almost half of the patients during convalescences could have been partially due to a dysregulated RAS. (3) Conclusions: Standard tests for routine patient care in Long COVID come often back normal. We suggest that the measurement of selected members of the RAS such as ACE and angiotensin II or the use of our BK degradation assay could identify Long COVID candidates. Clinical studies are required to test this hypothesis.

## 1. Introduction

### 1.1. The Renin–Angiotensin System and COVID-19

It was recognized early on during the corona virus infectious disease 2019 (COVID-19) pandemic that comorbidities such as hypertension and cardiovascular disease present major risk factors for severe illness and fatal outcomes. Two facts caused the spotlight in COVID-19 research on the renin–angiotensin system (RAS): (1) It is critical to retain blood pressure homeostasis, and according to experimental and clinical data, severe acute respiratory syndrome coronavirus type 2 (SARS-CoV-2) infection promotes a rise in blood pressure during the acute phase of infection [1]. (2) The RAS’ counter-regulatory process involves angiotensin-converting enzyme 2 (ACE2), the functional receptor of SARS-CoV-2 [2,3]. 

### 1.2. Dysregulated Serum ACE and CPN Activity in Convalescent COVID-19 Patients

**Figure 1:**
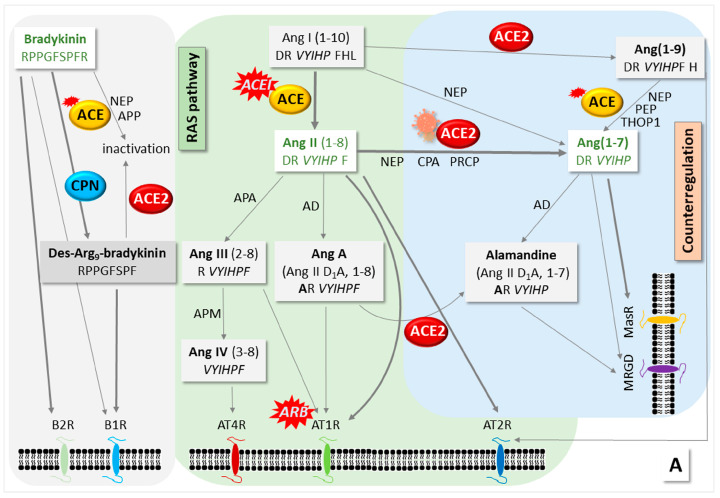

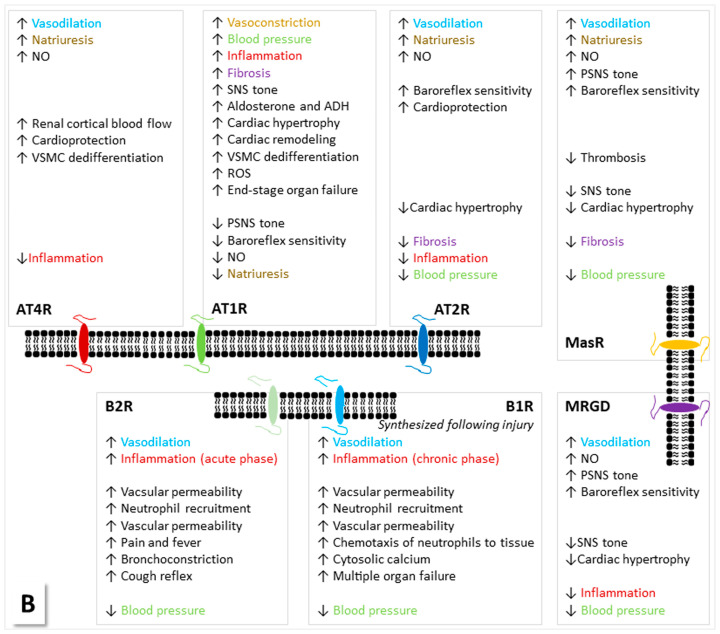
The connection of the kallikrein–kinin system (KKS) and the renin–angiotensin system (RAS) with respect to intervention by SARS-CoV-2. (**A**) Bioactive peptides, their receptors and (**B**) their functions. For involved proteins, see Supplementary Figure S1. The KKS and RAS are connected by the action of the angiotensin-converting enzyme (ACE), which deactivates bradykinin (BK) and cleaves angiotensin (Ang) I to Ang II. Vasoactive BK is formed from kininogen by the action of kallikrein. The latter needs the Hageman factor to be cleaved from its precursor, and it also influences the RAS by catalyzing the cleavage of prorenin to renin, which, in turn, assists the formation of Ang I from angiotensinogen. The generation of Ang II ensures blood pressure homeostasis and is counter-balanced by ACE2, which cleaves Ang II. Carboxypeptidase N (CPN) degrades BK to des-Arg9-BK, which can be deactivated by ACE2. In the classical RAS, Ang II binds to its receptors AT1R and AT2R with opposite effects on, among other functions, blood pressure and inflammation. The main antagonist to the classical RAS is ACE2, which removes Ang II by cleavage to Ang (1–7); this peptide binds to the Mas receptor (MasR), thereby reducing blood pressure. BK acts via receptors B1 and B2. CPN cleaves BK and thus changes receptor specificity from B2R to B1R [7,8,9]. ACE inactivates BK, and ACE2 cleaves des-Arg9-BK. More processes involving several other enzymes such as neutral endopeptidase/neprylisin (NEP) have been described in addition to the main pathways. Positions where inhibitors (ACEIs/ARBs) act are marked. The figures were assembled based on information collected in refs. [5,6] and reviews [2,3,10,11,12]. AD—aspartate decarboxylase, APA—aminopeptidase A, APN—alanyl aminopeptidase N, CPA—carboxypeptidase A, MRGD—Mas-related G protein-coupled receptor member D, NO—nitric oxide, PEP—prolyl endopeptidase, PSNS—parasympathetic nervous system, ROS—reactive oxygen species, SNS—sympathetic nervous system, THOP1—thiocyanate oligopeptidase, VSMC—vascular smooth muscle cells.

In earlier work, we have studied RAS components in sera of hospitalized COVID-19 and convalescent patients with both a bradykinin (BK) degradation assay [4] and high-definition mass spectrometry-based expression analysis (see publications for details [5,6]). BK is a target of angiotensin-converting enzyme (ACE), a critical enzyme of the RAS, and carboxypeptidase N (CPN) a member of the kallikrein-kinin system (KKS) [7,8]. RAS and KKS are connected by ACE activity (Figure 1, Supplementary Figure S1). We found that the degradation of the labelled form of BK (dabsylated BK—DBK) was generally impaired in hospitalized patients. CPN activity was significantly reduced. The DBK cleavage product generated by ACE, fragment 1–5 (DBK1–5), was increased in critically ill patients and strongly correlated with clinical heart and liver parameters [5]. Experimental values returned to normal levels during convalescence in the majority of patients, but almost half of the probands showed similar results as measured in hospitalized patients. At the time of our experiments, persisting post-COVID-19 symptoms were not fully recognized. We here hypothesize, based on the current literature, that dysregulated CPN and ACE serum activity could be an indication for the development of Long COVID in some patients. 

Mass spectrometric expression analysis of undepleted patient sera revealed significantly different protein profiles of hospitalized and convalescent patients as well as healthy probands as expected, but it also showed six distinct subgroups for the first cohort [6]. The protein profiles distinguished patients of different disease severity and pathophysiology. Thereby, one group represented the youngest and not severely ill patients, while another encompassed critically ill patients, who were, on average, older than 55 and overweight. The groups could be arranged according to disease severity; a correlation with age, body mass index, SAPS II (Simplified Acute Physiology Score [13]), and the number of abnormal laboratory parameters was then evident. The protein profiles indicated the presence of at least two major pathophysiological schemes differing in KKS/RAS activity and possibly defects in the complement alternative pathway [6]. Likely, also seroconversion played a role, because other authors have demonstrated that it stages COVID-19 patients into distinct pathological states [14]. Gender was not critical in our study which consisted mostly of male probands [5,6], and, on a side note, estrogen has been shown to inhibit inflammation and immune response in COVID-19 [15]. This may be one reason for the fact that males have been more affected by severe COVID-19 progression than females. 

Remarkably, the serum concentration of the precursor protein in the RAS, angiotensinogen, correlated with disease severity in hospitalized patients indicating that angiotensin (Ang) II-stimulated signaling was dysregulated. A clear distinction based on co-morbidities was not observed for the subgroups of hospitalized patients. In fact, hypertensive and antihypertensiva-taking patients were found in all groups. Interestingly, we observed that none of the hospitalized patients taking ACE inhibitors (ACEIs) or angiotensin receptor blockers (ARBs) died in contrast to half of the untreated hypertensive patients [5]. Indeed, studies have been published which provided evidence that ACEI/ARB medication is not enhancing COVID-19 symptoms and that it can even be protective [2,3]. We have followed the research into the topic with great interest, because this medication has an impact on the results of our BK reporter assay [4]. 

Below, we briefly summarize the current knowledge about the RAS in the light of our own findings. We discuss the data from our study in the context of recent information regarding the so-called Long COVID syndrome [16] and demonstrate that the measurement of members of the RAS may have diagnostic potential in that respect. 

## 2. Review and Discussion

### 2.1. The RAS 

The main product of the classical RAS is Ang II, which is formed from angiotensinogen with the assistance of renin and ACE. The binding of Ang II to its receptors leads to effects such as elevated blood pressure and disturbed renal water sodium balance. The counter-regulatory arm of the RAS involves ACE2, which cleaves Ang II and generates the products Ang (1–9), Ang (1–7) and alamandine, with opposite effects to those of Ang II. ACE2 also serves as a virus entry receptor during SARS-CoV-2 infection, leading to dysregulation. Another counter-balance to the RAS is the KKS, in which BK production contributes to blood-pressure-decreasing effects [7,8]. BK is inactivated by ACE [10]. CPN also degrades BK to des-Arg9-BK, which, in turn, can be deactivated by ACE2 [11]. We present Figure 1 and Supplementary Figure S1 as overviews highlighting the connection of the KKS and the RAS (for recent reviews, see [2,3,9,17]).

Considering these main processes involving the inverse relationship between ACE and ACE2, the use of ACEIs/ARBs has been proposed to counteract the inflammatory effects of COVID-19 infection by shifting metabolic activity away from the AT1 receptor pathway toward the AT2/Mas receptor pathways, but these inhibitors may potentially also increase ACE2 activity [18]. The corresponding pathophysiological processes upon viral infection are far from clear. Higher levels of ACE2 may increase one’s susceptibility to COVID-19 by allowing more of the virus into cells, but having more ACE2 could also be organ-protective [19].

### 2.2. Antihypertensive Drugs ACEI/ARB and COVID-19

Many papers suggest that an imbalanced RAS may be associated with severe COVID-19 progression (see references [2,3,20,21,22] and an extensive overview of scientific results up to the year 2021 in the supplement to reference [5]). There were controversial discussions in the general public early on concerning potential dangers in the use of antihypertensive medication in COVID-19 [20,23]. These resulted partially from studies which associated higher in-hospital mortality or a higher risk of hospital admission with the use of ACEIs/ARBs [24,25,26]. However, by now, there is consensus that ACEI/ARB treatment should be continued in the presence of SARS-CoV-2 infection [2,3,18,24]. Retrospective analyses found no evidence for a positive correlation of the use of ACEIs/ARBs with COVID-19 severity [27], and some authors even reported an apparent reduction in the risk of mortality [21,28]. A review of almost eight million health records from the Providence Health System (2020–2021) [18] detected an association between a reduced risk of COVID-19 infection/complications and ACEI/ARB use. Another study on hypertensive patients specified a connection of the use of ACEIs/ARBs prior to intensive care and a reduced risk of in-hospital mortality, but it measured a prolonged hospital stay [29]. A 2023 meta-analysis among East-Asian patients reported reduced mortality and, in contrast, a shorter duration of hospital stay, especially for females [30]. The authors of a meta-analysis involving more than a million hypertensive people also associated the use of ACEIs/ARBs with a lower risk of hospitalisation, intubation or death, but they did raise serious concerns about the quality and bias of clinical studies regarding COVID-19 patients [31]. 

Meanwhile, the single-cell sequencing of airway samples showed that patients with hypertension had a distinct inflammatory predisposition of immune cells that correlated with critical COVID-19 progression [32]. The study found that both ACEIs and ARBs reduced the risk for severe illness, but ACEIs did so much more than ARBs: ACEI treatment seemed to dampen hyperinflammation and increase cells’ intrinsic antiviral responses and almost abolished the risk of severe disease. ARBs, however, only slightly reduced it. The authors explained their observations with the decrease in Ang(1–7) upon SARS-CoV-2 infection, which could be restored by ACEI, but not ARB treatment [32] (for positions in the RAS, where the inhibitors act, see Figure 1A). Other researchers suggested that ARB might be superior to ACEI for the treatment of hypertensive COVID-19 patients, because ACEIs do not inhibit non-ACE-mediated Ang II production, ACE-induced BK may instigate acute respiratory disease syndrome, and ARB alleviates sputa production and inflammation and attenuates lung fibrosis [10,33]. These ideas have thus been disproven experimentally. In summary, a substantial amount of COVID-19 research is currently being directed towards the counter-regulatory RAS, and in particular, the Ang(1–7)-MasR axis; several clinical trials are underway [2,3,34].

### 2.3. Long COVID

It became apparent during the pandemic that some patients did not recover well from the disease. In fact, at least 10% of SARS-CoV-2 infections continued with symptoms on multiple organs (heart, lung, immune system, pancreas, gastrointestinal tract, neurological system, kidney, spleen, liver, blood vessels, reproductive system and skin) with an immense impact on both personal wellbeing and the healthcare system [16]. Through the machine learning analysis of over 137 symptoms and conditions from electronic health record data from the National Patient-Centered Clinical Research Network, four subphenotypes were identified, involving cardiac and renal (~33%); respiratory, sleep and anxiety (~33%); musculoskeletal and nervous system (~24%); and digestive and respiratory system (~10%) sequelae [35]. Symptoms such as myalgic encephalomyelitis/chronic fatigue syndrome and postural orthostatic tachycardia syndrome were already known from other viral-onset illnesses [16]. Long COVID affects both adults and children and predominates in non-hospitalized patients with mild acute illness, but symptoms are typically resolved within one year according to a recent study from Israel [36]. For a comprehensive overview of Long COVID regarding major findings, mechanisms and recommendations, the interested reader is directed to a timely 2023 review on the topic [16].

The post-acute sequelae of the infection are persistent, exacerbated or newly incident and thus difficult to diagnose and treat. Standard tests in routine patient care often come back normal, and it takes symptom-specific testing, of which, many providers are not aware [35]. As a result of the high complexity of Long COVID symptoms, the knowledge of specialists (cardiologists, neurologists, dermatologists, etc.) is required (for a list of available diagnostic tools and treatment options, see reference [16]). Therefore, the increasingly established treatment centers for Long COVID patients take an interdisciplinary approach.

### 2.4. Is Reduced BK Serum Degradation in Convalescent Patients Indicative of Long COVID?

In our earlier study [5,6], we detected convalescent patients whose sera degraded dabsylated BK as poorly as the hospitalized patients. At the time of our experiments, there was still little scientific data regarding the symptoms of complex Long COVID. We thus revisited the data with the hypothesis that the DBK assay could indicate Long COVID candidates. Unfortunately, we do not have follow-up data for the patient cohort regarding their symptoms months after the acute disease, so we cannot substantiate our hypothesis. In Supplementary Figure S2, we set the experimental data (results of the DBK degradation assay and protein intensities as obtained using mass-spectrometry-based expression analysis [5,6]) for patient groups in relation to those of healthy controls to illustrate the major findings. DBK serum degradation capacity and DBK1–8 formation were reduced in hospitalized patients, with no significant differences among subgroups. The lowest serum DBK degradation capacity was detected in the critically ill patients, along with the excessive formation of DBK1–5. Other hospitalized patients also showed poor values for DBK cleavage and only 60–80% of DBK1–8 formation compared to healthy controls, but the DBK1–5 levels were not conspicuous. These results indicated that in the first group—among other multi-system failures—and also both the RAS and the KKS were seriously compromised, while in the latter, ACE (as expressed by DBK1–5 formation) was unaffected. The other subgroups of hospitalized patients showed variations in these measures, indicating a different impact of CPN and ACE. Moreover, the serum concentration of angiotensinogen correlated with disease severity in hospitalized individuals; it was not conspicuous in convalescent probands. In general, the results of BK degradation experiments in the sera of convalescent patients were not reflected in the levels of abundant KKS/RAS-related serum proteins, which were normal [6]. 

Reduced BK serum degradation can result from both impaired serum CPN and dysregulated ACE activity [37,38]. In our earlier publication [6], we hypothesized that the former could be due to reduced CPN synthesis in the liver following organ impairment as a result of COVID-19. Possibly, this condition lasts much longer than the acute disease phase. The excessive DBK1–5 values in some convalescent patients, however, point to overactive ACE and the dysregulation of the RAS, as observed in acute COVID-19. The fact that ACE and CPN activity remains imbalanced in some individuals may assist research into Long COVID pathophysiology. Our low-tech assay can be of assistance in identifying these persons as it only requires a drop of capillary blood or serum [39]. 

## 3. Conclusions

Using BK degradation for measuring the protease (CPN and ACE) activity in patients’ sera, we noted a general reduction in the BK degradation capacity in hospitalized COVID-19 patients, but also in some convalescent patients. CPN activity was decreased and contributed to a large extent to the overall reduced serum BK degradation capacity. Increased values for the BK fragment DBK1–5, which is generated by ACE, were, however, also detected. These observations in some convalescent patients raised the question of whether the assay results could be indicative of Long COVID, in particular, because the DBK1–5 level significantly correlated with COVID-19 severity in hospitalized patients. This question can only be answered by dedicated clinical studies. They have potential, because the BK assay is a low-cost/low-tech procedure based on thin-layer chromatography [4] that can be performed in any laboratory and only requires small samples [39]. If its results truly indicate Long COVID candidates, it might be a valuable tool to speed up patient diagnosis and therapy. Other components of the RAS such as ACE, Ang II and Ang(1–7) could also be tested to obtain a deeper knowledge about the underlying pathophysiology. 

When designing the clinical studies, it is important to skillfully assemble patient cohorts with regard to co-morbidities. Diseases like metabolic syndrome, in particular hypertension, directly involve the RAS, and they are known risk factors in this virus infection. Care must be taken to have sufficiently large cohorts of untreated, ACEI- and ARB- (or otherwise-) treated hypertensive patients to be able to draw significant conclusions, because the medication influences the assay results. In fact, we have learned from the serum proteomics experiments that multiple parameters other than those typically considered during the assembly of patient cohorts (age, gender and co-morbidities) may influence the results. We detected six distinct groups among the hospitalized patients, which were distinguished by their serum protein profiles. These groups seemed to represent subsets of different pathophysiologies (e.g., different levels of RAS, KKS and complement involvement). A correlation with co-morbidities such as hypertension or diabetes could, however, not be detected. In fact, hypertensive patients were present in all groups. Clearly, such subgroups should not be combined in subsequent data mining. The results would only show the most striking differences among the major groups of probands, and subtle differences would go unnoticed. These observations may be one reason for the bias seen in COVID-19 research [31], and they beg the question of how to best assemble study cohorts. More often than not, researchers are limited by the availability of eligible patients. An increase in participant numbers and the formation of sufficiently large cohorts can help so that subgroups can be later evaluated with sufficient power.

In summary, we have revisited earlier proteomics data and results on serum protease activity obtained with BK as a reporter peptide from hospitalized and convalescent COVID-19 patients [5,6]. We found that the BK degradation in patient sera can be abnormal in convalescent patients. As our assay reports on CPN and ACE activity, it may be indicative of persisting liver problems and/or a dysregulated RAS. In light of the general acceptance of Long COVID, we hypothesize that monitoring of members of the RAS such as ACE and Ang II may identify Long COVID candidates. Imbalances in the RAS, characterized by increased ACE activity and reduced ACE2 activity, could contribute to inflammation, vasoconstriction, oxidative stress and tissue damage observed in severe COVID-19 cases [2,3,8]. These imbalances may also have implications for the development of Long COVID symptoms. However, Long COVID is a very complex disease, which manifests in at least four subphenotypes [35], meaning that likely not all will be detectable in the same manner; probably, only the cardiac and renal subgroup will show a response. Clinical studies are required to test the hypothesis.

## 4. Reference Information

This paper discusses data generated in two earlier studies [5,6] of 45 hospitalized patients with laboratory-confirmed SARS-CoV-2 infection, admitted to the University Hospital Münster and Marien-Hospital Steinfurt in Germany between March and June 2020, 26 individuals with laboratory-confirmed SARS-CoV-2 infection, who had recovered, and 8 healthy volunteers. The Ethics Committee of Münster University approved the study (AZ 2020-220-f-S and AZ 2020-210-f-S), and the procedures were in accordance with the Helsinki Declaration of 1975 as revised in 1983. For all patient and measurement details, see our earlier publications [5,6]. 

## Data Availability

Data have been made available in earlier publications [5,6].

## Supplementary Materials

The following supporting information can be downloaded at: https://www.mdpi.com/article/10.3390/life13071462/s1. Figure S1: Proteins involved in KKS and RAS, Figure S2: data.
