# Supplementary material for: The Renin–Angiotensin System in COVID-19: Can Long COVID Be Predicted?
Source: Life (Basel). 2023 Jun 28;13(7):1462. doi: 10.3390/life13071462 (PMC10381802; doi:10.3390/life13071462)
Supplement: Supplementary file 1 [file life-13-01462-s001.zip › life-2421066-supplementary.pdf]

life

MDPI

Communication

The Renin–Angiotensin System in COVID-19: Can Long COVID Be Predicted?

Simone König [1,*], Richard Vollenberg [2,†] and Phil-Robin Tepasse [2,†]

[1] IZKF Core Unit Proteomics, University of Münster, 48149 Münster, Germany
[2] Department of Medicine B for Gastroenterology, Hepatology, Endocrinology and Clinical Infectiology, University Hospital Muenster, 48149 Münster, Germany
* Correspondence: koenigs@uni-muenster.de
† These authors contributed equally to this work.

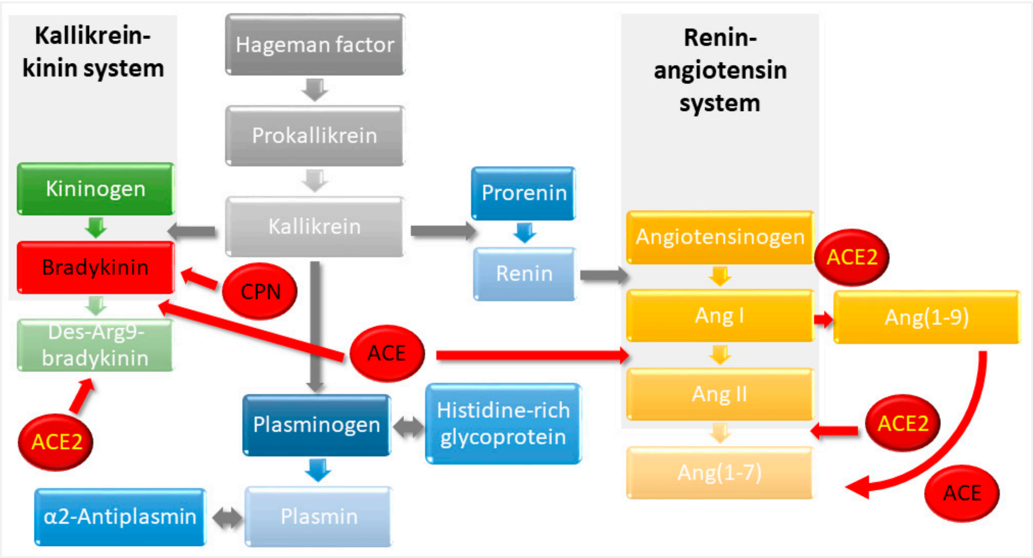

Figure S1. The connection of the kallikrein-kinin system (KKS) and the renin-angiotensin system (RAS) with respect to intervention by SARS-CoV-2. KKS and RAS are connected by the action of angiotensin-converting enzyme (ACE), which deactivates bradykinin (BK) and cleaves angiotensin (Ang) I to Ang II. Vasoactive BK is formed from kininogen by the action of kallikrein (KLKB1). The latter needs Hageman factor to be cleaved from its precursor. It also influences the RAS by catalyzing the cleavage of prorenin to renin, which assists the formation of Ang I from angiotensinogen. The generation of Ang II ensures blood pressure homeostasis and is counterbalanced by ACE2, which cleaves Ang II. KLKB1 can activate Hageman factor and plasminogen (PLG). Plasmin degrades fibrin, initiating fibrinolysis, and it is inhibited by α2-antiplasmin. Histidine-rich glycoprotein has roles in immunity, angiogenesis and coagulation and binds PLG. Carboxypeptidase N (CPN) degrades BK to des-Arg9-BK, which can be deactivated by ACE2. Modified from our earlier publication ([6]).

G	1	3	5	4	2	6	CoP-NV	CoP-DV
DBK1-9	>190	160-170	>190	130-160	130-160	>190	90-100	130-160
DBK1-8	60-80	60-80	60-80	>80	>80	<60	>80	60-80
DBK1-5	90-120	90-120	80-90	80-90	130-140	>160	90-120	130-140
AGT	50-60	100-130	140-150	140-150	181	>250	100-130	100-130
PLG	<60	>80	<60	<60	60-70	60-70	>80	>80
KLKB1	<20	>80	<20	<20	>80	40-50	>80	>80
HRG	<30	>80	<30	<30	>80	~40	>90	>90
KNG	<40	>90	40-60	40-60	>90	>90	>90	>90
F12	<40	>80	50-70	50-70	>80	>80	50-70	>80
SERPINF2	>700	400-500	100-200	100-200	300-400	300-400	300-400	300-400

Figure S2. Re-evaluation of earlier data ([6]; see publication for details).

Results for hospitalized patients (HoP, groups G1 to G6) and convalescent patients (CoP; CoP-NV – group with normal bradykinin serum degradation during convalescence, CoP-DV - with assay values close to those of HoP during convalescence): DBK degradation assay (top panel): measured intensities for the labelled peptide and its fragments following incubation with patient sera; HDMS expression analysis of patient sera – selected proteins (bottom panel). Average experimental values for each group were set in relation to those of the healthy cohort and expressed in percent. DBK1-9/ DBK – dabsylated bradykinin; DBK1-8/1-5 – DBK fragments 1-8 and 1-5, AGT – angiotensinogen, F12 - Hageman factor/complement factor 12, HRG - histidine-rich glycoprotein, KLKB1 – kallikrein, KNG – kininogen, PLG – plasminogen, SERPINF2 - α2-antiplasmin, G – group, HDMS - high-definition mass spectrometry. Coloring according to deviation from normal values (red – extreme values, green – values close to normal, yellow and orange – intermediate stages).
